# Identification and genomic analyses of a novel endophytic actinobacterium *Streptomyces endophytica* sp. nov. with potential for biocontrol of yam anthracnose

**DOI:** 10.3389/fmicb.2023.1139456

**Published:** 2023-04-04

**Authors:** Shuangqing Zhou, Yifan Zhou, Chengui Li, Wenqiang Wu, Yun Xu, Wei Xia, Dongyi Huang, Xiaolong Huang

**Affiliations:** ^1^Department of Pharmacognosy, College of Pharmacy, Guilin Medical University, Guilin, China; ^2^Department of Biotechnology, School of Life Sciences, Hainan University, Haikou, China; ^3^Department of Agronomy, College of Tropical Crops, Hainan University, Haikou, China

**Keywords:** *Streptomyces endophytica* sp. nov., anthracnose, endophytic actinobacteria, biological control, yam

## Abstract

Anthracnose disease caused by *Colletotrichum gloeosporioides* is one of the devastating diseases of yams (*Dioscorea* sp.) worldwide. In this study, we aimed to isolate endophytic actinobacteria from yam plants and to evaluate their potential for the control of yam anthracnose based on bioassays and genomic analyses. A total of 116 endophytic actinomycete strains were isolated from the surface-sterilized yam tissues from a yam orchard in Hainan Province, China. In total, 23 isolates showed antagonistic activity against *C. gloeosporioides*. An endophytic actinomycete, designated HNM0140^T^, which exhibited strong antifungal activities, multiple biocontrol, and plant growth-promoting (PGP) traits was subsequently selected to colonize in the tissue-cultured seedlings of yam and was tested for its *in vivo* biocontrol potential on yam anthracnose. The results showed that treatment with strain HNM0140^T^ markedly reduced the severity and incidence of yam anthracnose under greenhouse conditions. Morphological and chemotaxonomic analyses showed that strain HNM0140^T^ was assigned to the genus *Streptomyces*. Phylogenetic analysis based on the 16S rRNA gene sequences indicated that strain HNM0140^T^ formed a separate cluster together with *Streptomyces lydicus* ATCC 25470^T^ (99.45%), *Streptomyces chattanoogensis* NRRL ISP-5002^T^ (99.45%), and *Streptomyces kronopolitis* NEAU-ML8^T^ (98.97%). The phylogenomic tree also showed that strain HNM0140^T^ stably clustered with *Streptomyces lydicus* ATCC 25470^T^. The ANI and dDDH between strain HNM0140^T^ and its closest related-type species were well below the recommended thresholds for species demarcation. Hence, based on the phylogenetic, genomic, and phenotypic analyses, strain HNM0140^T^ should represent a new streptomycete species named *Streptomyces endophytica* sp. nov. Genomic analysis revealed that strain HNM0140^T^ harbored 18 putative BGCs for secondary metabolites, some PGP-related genes, and several genes coding for antifungal enzymes. The presented results indicated that strain HNM0140^T^ was a promising biocontrol agent for yam anthracnose.

## Introduction

Yam (*Dioscorea* spp.) is a very important tuber crop that is widely cultivated in both tropical and sub-tropical regions around the world. Yam tubers are widely consumed as a valuable source of food across the globe (Egesi et al., 2007). Chinese yam (*Dioscorea opposita* Thunb.) is among the most widely cultivated yam species in China both as a traditional medicine and as a nutritious food source (Li et al., 2014). However, anthracnose disease, caused by *Colletotrichum gloeosporioides*, is one of the devastating diseases of yam and seriously threatens yam production worldwide (Abang et al., 2003). The pathogenic fungus can not only infect all parts of yam plants but also affect all growth stages of yam, which results in serious yield losses, especially in susceptible genotypes (Nwadili et al., 2017). Currently, the anthracnose disease of yam has been controlled mainly using chemical methods (Onyeka et al., 2006). However, chemical fungicides have been reported to not always be effective against anthracnose disease, especially during the rainy season (Ntui et al., 2021). Meanwhile, their frequent use has caused deleterious effects, including the development of fungicide-resistant pathogens and damage to the environment (Onyeka et al., 2006). Thereby, it is important to call for investigating alternative control measures including biological control, which is favored as an ecofriendly alternative to chemical fungicides for its decreasing reliance on the dangerous use of chemicals and its lesser cost (Okigbo, 2005).

As an effective tool of biological control, microbial antagonism has been attracting the attention of researchers in recent years, some of whom have found a novel method for selecting antagonists against anthracnose diseases in a variety of crops, such as strawberry, chili, and rubber (Thilagam and Hemalatha, 2019; Gu et al., 2020; Marian et al., 2020). Actinomycetes, as prolific producers of antibiotics, have been studied extensively as promising antagonists to control plant pathogens (Doumbou et al., 2001; Solanki et al., 2016; Zhang et al., 2020). Many previous studies have suggested that actinomycetes inhabit inside plant tissues as endophytes (Govindasamy et al., 2014), some of which have been used for effective biocontrol and as PGP agents (Shimizu, 2011; Singh and Dubey, 2018; Gao et al., 2021). However, only a few studies have reported the utilization of actinomycetes to manage anthracnose diseases of yam (Soares et al., 2006; Palaniyandi et al., 2011, 2013, 2016).

Thus, the present study was designed to isolate endophytic actinobacteria from yam plants and to evaluate their potential for the biocontrol of yam anthracnose. A novel endophytic actinobacterial strain of HNM0140^T^ isolated from the root of yam displayed a strong antifungal activity against *C. gloeosporioides*, which was isolated from naturally infected yam leaves, and its taxonomic status was established through a polyphasic taxonomic approach. We identified strain HNM0140^T^ as a novel actinobacterial species named *Streptomyces endophytica* sp. nov. and evaluated its biocontrol ability against yam anthracnose. Moreover, the complete genome of strain HNM0140^T^ was also sequenced and analyzed for further understanding of its biocontrol potential at genomic levels.

## Materials and methods

### Isolation of endophytic actinobacteria

Plant materials (leaf, stem, and root samples) of healthy *Dioscorea opposita* Thunb. plants were collected from a yam orchard in Danzhou City (19°31′N, 109°35′E) of Hainan Province, China. The surface-sterilized procedures of plant materials were performed according to the method previously described (Huang et al., 2012). A total of 1 g of surface-sterilized plant materials was then cut into small fragments and homogenized in 10 ml of sterile phosphate-buffered saline (PBS). The resultant homogenates (0.1 ml) were plated onto three isolation media: humic acid–vitamin agar (Hayakawa and Nonomura, 1987), tap water–yeast extract agar (Crawford et al., 1993), and IMA-2 agar (Shimizu et al., 2000) supplemented with 50 mg/L of actidione, 50 mg/L of nystatin, and 100 mg/L of potassium dichromate (K_2_Cr_2_O_7_). The plates were incubated at 28°C for 28 days. Presumptive actinobacterial colonies that appeared on the isolated plates were picked and streaked onto ISP2 agar (Shirling and Gottlieb, 1966) and cultured at 28°C for 1–2 weeks.

### *In vitro* antagonistic bioassay

Antagonistic activities of the endophytic isolates against *C. gloeosporioides*, a major pathogen of yam, were evaluated on potato dextrose agar (PDA) plates using the dual culture *in vitro* assay as described previously by Palaniyandi et al. (2011). All isolates of endophytic actinobacteria were streaked onto PDA plates and incubated at 28°C for 3 days. Mycelial disks of 5-mm diameter of *C. gloeosporioides* were placed onto the described PDA plates 5 cm away from each actinobacterial colony. The phytopathogenic fungi were separately incubated on non-inoculated PDA plates as a control. After culturing at 28°C for 7 days, the plates were examined for the inhibition zone between the pathogen and the actinobacterial isolate according to the method described previously (Crawford et al., 1993). Endophytic actinobacteria, designated as strain HNM0140^T^, were obtained from the root of *D. opposita* Thunb., which was selected for further studies on the basis of its strong antifungal activity against *C. gloeosporioides*. Strain HNM0140^T^ was also tested for its antifungal activities against other phytopathogenic fungi, namely, *Fusarium oxysporum* f. sp. cubense race 4 from banana, *Magnaporthe oryzae* from rice, *Lasiodiplodia theobromae* from mango, *Neoscytalidium dimidiatum* from pitaya, *Colletotrichum capsici* from chili, and *Stagonosporopsis cucurbitacearum* from watermelon. These aforementioned pathogens were provided by the College of Life Sciences, Hainan University, Haikou, China.

### Characterization for biocontrol and PGP traits

The production of siderophore and protease by strain HNM0140^T^ was detected as described in a previous study (Naik et al., 2008). The β-1,4-glucanase activity of strain HNM0140^T^ was screened according to a previously described method (Palaniyandi et al., 2011). The chitinase activity of strain HNM0140^T^ was assessed using colloidal chitin agar medium as described in a previous study (Hsu and Lockwood, 1975). The nitrogen-fixing ability of strain HNM0140^T^ was assessed using N-free semisolid JNFb medium (Döbereiner et al., 1995). The phosphate solubilization activity of strain HNM0140^T^ was detected as described in a previous study (Hamdali et al., 2008). The ACC deaminase activity and indole acetic acid production (IAA) were evaluated as described previously (El-Tarabily, 2008). The ability of the PGP of strain HNM0140^T^ was assessed by a plate-based bioassay according to the modified method described previously (Palaniyandi et al., 2011). An agar block (6-mm diameter) containing mycelia of the strain HNM0140^T^ was placed in the center of plates containing MS medium (Murashige and Skoog, 1962) with 0.8% (w/v) agar and grown at 28°C for 3 days. Twenty stratified *Arabidopsis* Col-0 seeds were placed 3 cm away from the strain HNM0140^T^ culture on the MS plate and incubated in a growth chamber at 25°C for 7 days. The MS plate inoculated with *Arabidopsis* seeds only was used as the control. After 7 days, the root lengths and total fresh weight of the plants were measured.

### Colonization of strain HNM0140^T^ on tissue-cultured seedlings of yam

*In vitro* plantlets of yam (cultivars: *D. opposita* Thunb.) were raised from nodal explants for 1 month in 75 × 105-mm glass flasks using MS medium supplemented with bacto agar (0.8%) and sucrose (3%). These explants were incubated in a growth chamber at 25°C under a 16-h photoperiod for another month. The resultant seedlings were prepared for the following colonization test. The colonization of strain HNM0140^T^ on tissue-cultured seedlings of yam was examined using the method described previously by Shimizu et al. (2001). Strain HNM0140^T^ was inoculated into 150 ml of ISP2 broth and cultured on a shaker (180 rpm) at 28°C for 3 days. Then, 2 ml of the mycelial suspension (5–6 × 10^6^ CFU/ml) was added to the surface of MS medium in glass flasks supporting yam seedlings. For the control, 2 ml of ISP2 broth was also spread on the surface of the MS medium. These seedlings, inoculated and non-inoculated with strain HNM0140^T^, were grown for 2 weeks. Then, the seedlings were taken from the flasks and were cut into small fragments at each node. These fragments were placed on ISP2 agar and incubated at 28°C for ~14 days. For each seedling, the colonized strain was re-isolated and re-identified by comparing the morphological characteristics and the 16S rRNA gene sequence with those of strain HNM0140^T^. The detached leaves were also observed by scanning electron microscopy (S-3000N, Hitachi, Japan) according to the methods previously described (Shimizu et al., 2009).

### *In vivo* biocontrol trial

To validate its suitability as a biocontrol agent, the seedlings of yam, treated and untreated with strain HNM0140^T^ in flasks as previously, were transferred into a tray (50 × 25 × 7 cm^3^) containing autoclaved soil. Then, the trays were transferred into a large bioclimatic chamber, and the seedlings were acclimatized at 30/25°C day/night temperature and 12-h photoperiod for 2 weeks. At the end of the acclimatization period, the seedlings were then spray-inoculated with *C. gloeosporioides* spore suspension (2 × 10^6^ spores/ml) using an atomizer at the rate of ~5 ml per seedling. Each experiment was replicated three times and eight plants were used for each replicate. These inoculated seedlings were transferred into a humid chamber (RH 95%, 28°C, dark) for 24 h and then placed in a greenhouse with 85% relative humidity under a 12-h photoperiod at 28°C for 2 weeks. The yam seedlings were monitored daily for anthracnose, and the disease incidence and severity were assessed after 2 weeks of inoculation of *C. gloeosporioides*. The disease incidence was calculated as the percentage of diseased leaves among the total leaves studied in each tray (Palaniyandi et al., 2011). The disease severity index was calculated according to the methods described previously (Simons and Green, 1994). The disease severity of each seedling was scored using a 0–6-point scale on the basis of the percentage of the whole seedling area affected by anthracnose, where 0 = 0%, 1 = 1%, 2 = 2%, 3 = 3–9%, 4 = 10–24%, 5 = 25–50%, and 6 >50% (Onyeka et al., 2006).

### Phylogenetic analyses of strain HNM0140^T^

Genomic DNA was extracted from strain HNM0140^T^ using the Wizard^®^ Genomic DNA Purification Kit (Promega Corp.). PCR amplification and sequencing of the 16S rRNA gene of strain HNM0140^T^ were performed as described by Huang et al. (2012). The 16S rRNA gene sequence of strain HNM0140^T^ was aligned with those of the type strains by the EzTaxon-e server (Yoon et al., 2017). Phylogenetic trees were generated using neighbor-joining (Saitou and Nei, 1987), minimum-evolution (Rzhetsky and Nei, 1992), and maximum-likelihood (Felsenstein, 1981) methods from the MEGA 7.0 software (Kumar et al., 2016). Evolutionary distances were calculated using the Kimura 2-parameter model (Kimura, 1980). Confidence interval levels of the branch nodes were studied by a bootstrap analysis of 1,000 replications (Felsenstein, 1985).

### Genomic analyses of strain HNM0140^T^

The whole-genome sequencing and assembly of strain HNM0140^T^ were carried out as described by Zhou et al. (2018). Protein-coding gene prediction was carried out by Glimmer v3.02 software (Delcher et al., 2007), whereas rRNA and tRNA were predicted using Infernal 1.1 (Nawrocki and Eddy, 2013) and tRNAscan-SEv1.31 (Lowe and Eddy, 1997) software, respectively. Their functions were annotated using the databases of clusters of orthologous genes (COGs), the Gene Ontology (GO), and the Kyoto Encyclopedia of Genes and Genomes (KEGG). The secondary metabolite gene cluster prediction was performed using the online antiSMASH 6.0 software (Blin et al., 2021).

### Phylogenomic analyses of strain HNM0140^T^

The phylogenomic tree of the whole genome was constructed on the Type Strain Genome Server (TYGS) (Meier-Kolthoff et al., 2022). Digital DNA–DNA hybridizations (dDDHs) were calculated with a Genome-to-Genome Distance Calculator (GGDC) v3.0 (Meier-Kolthoff et al., 2022). A comparison of the average nucleotide identity (ANI) values was performed using the JSpeciesWS Online Service (Richter et al., 2016).

### Phenotypic characteristics

Morphological characteristics of strain HNM0140^T^ were observed by scanning electron microscopy (S-3400N, Hitachi, Japan) after incubation for 14 days at 28°C on ISP2 agar. Cultural characteristics of strain HNM0140^T^ were determined after 2 weeks at 28°C using standard ISP 1–ISP 7 media (Shirling and Gottlieb, 1966). Colors of the colony and diffusible pigments on various media were determined by a comparison with the ISCC–NBS color charts (Kelly, 1964). The effects of different temperatures, pH values, and sodium chloride (NaCl) concentrations on growth were observed using ISP2 medium as the basal medium after incubation at 28°C for 14 days as described in a previous study (Huang et al., 2019). The carbon substrate and nitrogen source utilization was examined following the method of Shirling and Gottlieb (1966) and the method of Williams et al. (1983), respectively. Hydrolysis of aesculin, starch, and Tween 40, peptonization and coagulation of milk, and production of urease were tested as described in a previous study (Yokota et al., 1993).

### Chemotaxonomic characteristics

Cell biomass obtained after culturing strain HNM0140^T^ in shake flasks of ISP2 broth for 4–6 days at 28°C was used for chemotaxonomic analyses. The analyses of amino acids and sugars in whole-cell hydrolysates of strain HNM0140^T^ were performed as described previously by Lechevalier and Lechevalier (1980). Phospholipid and menaquinone analysis was performed according to the methods of Minnikin et al. (1984). Fatty acids of strain HNM0140^T^ were extracted and analyzed using the method of Sasser (1990).

## Results and discussion

### Isolation and antifungal activity of endophytic actinobacteria

**Figure 1:**
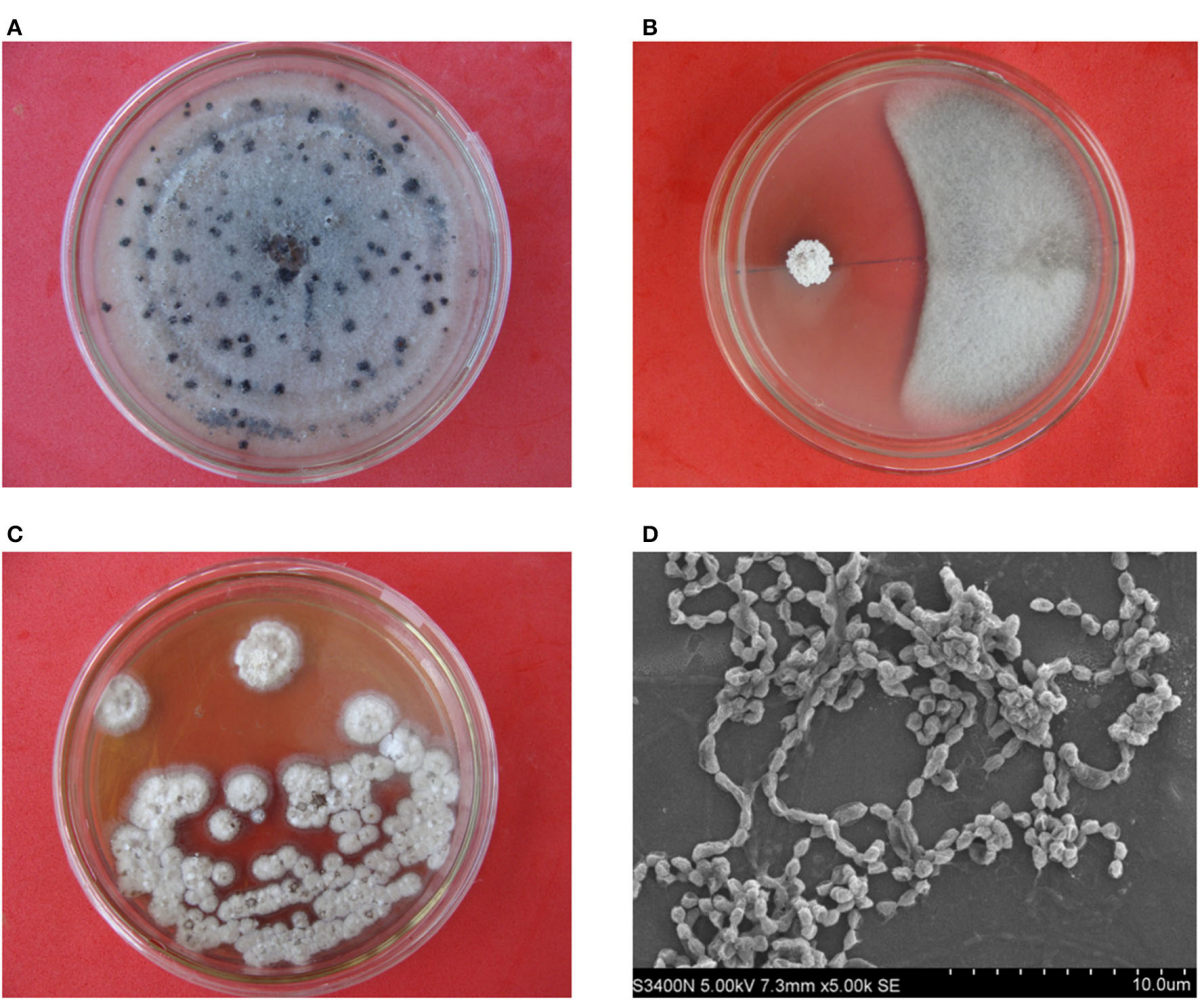
Antifungal activity of strain HNM0140^T^ against *C. gloeosporioides* on a PDA plate and morphological features of HNM0140^T^ colonies and spore chains. **(A)** *C. gloeosporioides* growth without antagonist challenge. **(B)** Inhibition of *C. gloeosporioides* growth by strain HNM0140^T^. **(C)** Morphological features of strain HNM0140^T^ colonies. **(D)** Scanning electron micrograph showing spore chains of strain HNM0140^T^.

A total of 116 endophytic actinobacteria were obtained from the different tissue samples of *D. opposita* Thunb. Out of them, 23 isolates showed more or less antifungal activity against *C. gloeosporioides* (Supplementary Table S1). Especially, strain HNM0140^T^ exhibited a strong antagonistic activity against *C. gloeosporioides* (Figures 1A, B). Consequently, strain HNM0140^T^ was selected further for biocontrol of yam anthracnose diseases caused by the pathogen.

### Biocontrol and PGP traits of strain HNM0140^T^

The various biocontrol and PGP traits of strain HNM0140^T^ are summarized in Table 1. Strain HNM0140^T^ showed a strong antagonistic activity on *C. gloeosporioides, C. capsici*, and *F. oxysporum* f. sp. *cubense* race 4, only a moderate activity on *M. oryzae* and *S. cucurbitacearum*, and a weaker activity on *L. theobromae* and *N. dimidiatum* (Table 1). Strain HNM0140^T^ exhibited positive results for the production of protease, β-1,4-glucanase, chitinase, and siderophore. The strain also showed activity to growth in N-deficient media, phosphate solubilization, IAA synthesis, and ACC deaminase (Table 1). The strain was also capable of inducing multiple lateral root development of *Arabidopsis* seedlings with more root hairs, whereas the control seedlings exhibited longer roots with few lateral roots and fewer root hairs (Supplementary Figure 1).

**Table 1:** Traits of biocontrol and PGP of strain HNM0140^T^.

**Test**	**Result**
**Antifungal activity** ^**a**^	
*Colletotrichum gloeosporioides*	+++
*Fusarium oxysporum* f. sp. *cubense* race 4	+++
*Magnaporthe oryzae*	++
*Lasiodiplodia theobromae*	+
*Neoscytaldium dimidiatum*	+
*Colletotrichum capsici*	+++
*Stagonosporopsis cucurbitacearum*	++
**Biocontrol traits** ^**b**^	+
β-1,4-glucanase production	+
Chitinase production	+
Protease production	+
Siderophore production	+
**Plant growth-promoting traits** ^**b**^	
Growth on N free media	+
Phosphate solubilization	+
IAA production	+
ACC deaminase activity	+
Arabidopsis growth	+

a +++, zone of inhibition ≥2.0 cm; ++, zone of inhibition 2.0–1.0 cm; +, zone of inhibition < 1.0 cm; –, no zone of inhibition.

b +, presence of activity; –, absence of activity.

### Colonization of strain HNM0140^T^ on tissue-cultured seedlings of yam

**Figure 2:**
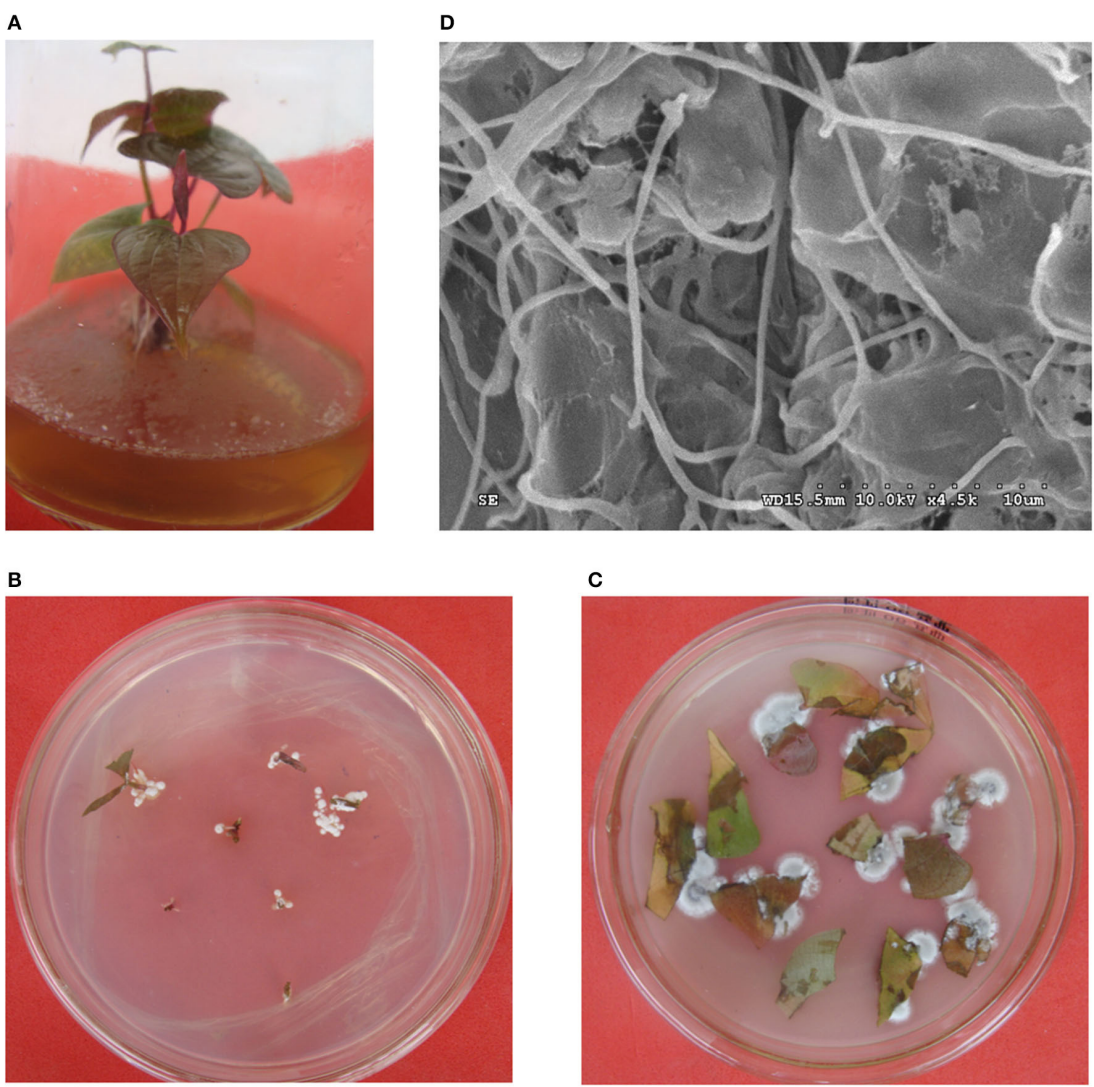
Colonization of strain HNM0140^T^ on tissue-cultured seedlings of yam. **(A)** Strain HNM0140^T^-colonized seedling. **(B)** Re-isolation of strain HNM0140^T^ from segments of stems of tissue-cultured seedlings of yam. **(C)** Re-isolation of strain HNM0140^T^ from segments of leaves of tissue-cultured seedlings of yam. **(D)** Scanning electron micrograph of the surface of leaves of tissue-cultured seedlings of yam after colonization of strain HNM0140^T^.

The results for the colonization test of strain HNM0140^T^ showed that the strain was successfully able to grow on MS medium (Figure 2A), but only one type of mycelial colony that had the same morphological characteristics with strain HNM0140^T^ was expanding from the roots, stems, and leaves of inoculated tissue-culture seedlings of yam within 2 weeks after placing their fragments on ISP2 agar (Figures 2B, C). The re-isolated strains from fragments were analyzed using 16S rRNA gene sequencing, and the results suggested that these strains were 100% identical to the original strain HNM0140^T^. At the same time, abundant mycelia were observed by SEM on the leaves from seedlings treated with strain HNM0140^T^ (Figure 2D). The results indicated that strain HNM0140^T^ was endophytic.

### *In vivo* biocontrol trial

**Figure 3:**
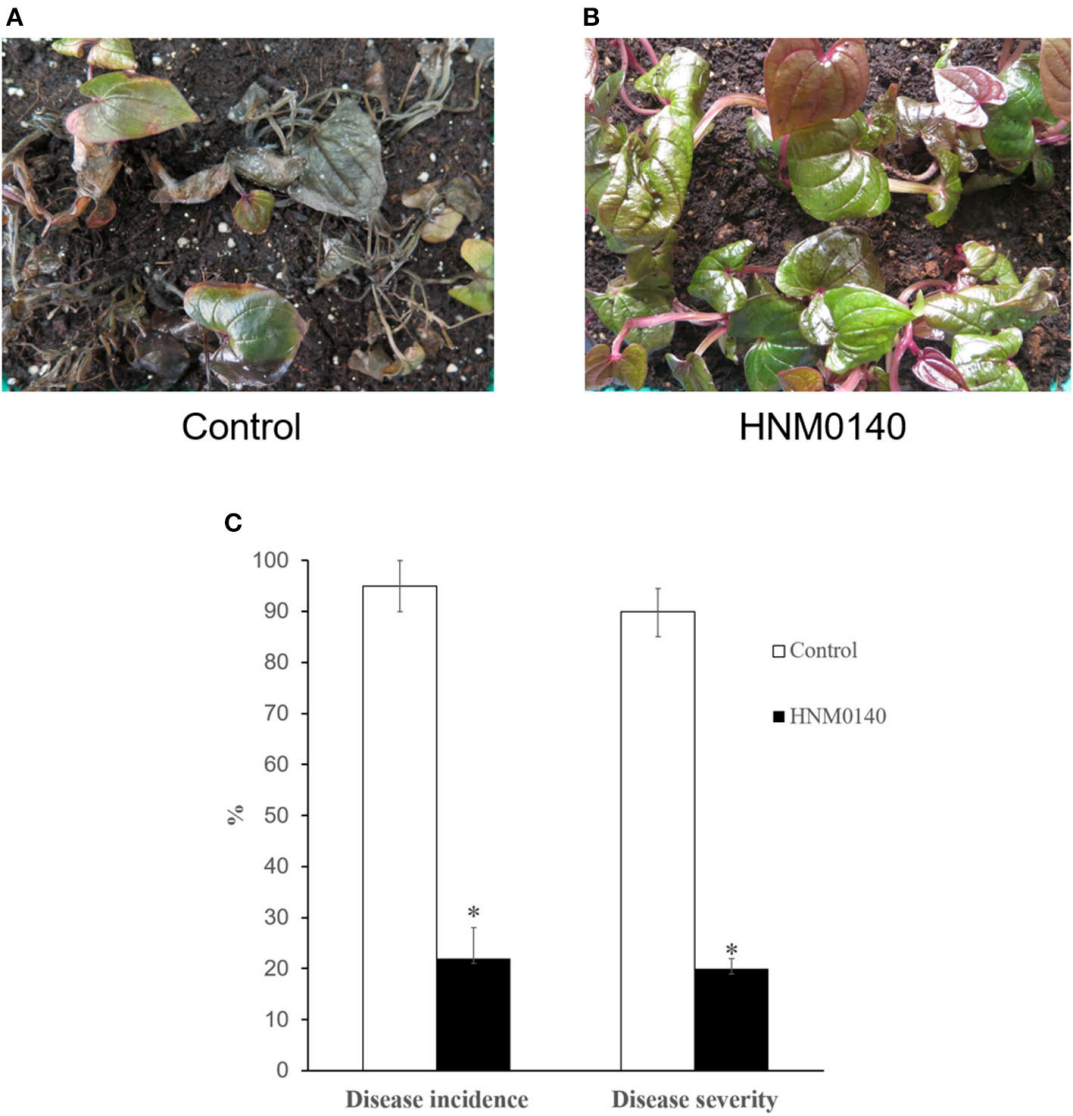
Effect of strain HNM0140^T^on yam anthracnose under greenhouse conditions. **(A)** Anthracnose symptoms on non-treated yam plants 14 days after inoculation with *C. gloeosporioides*. **(B)** Anthracnose symptoms on treated yam plants 14 days after inoculation with *C. gloeosporioides*. **(C)** Differences in disease incidence and severity of anthracnose of the treatment relative to the non-treated plants. Asterisk indicates significance in treated than in the non-treated category according to Fisher's protected LSD test at *P* = 0.05.

Anthracnose symptoms in control yam seedlings were first observed after 3 days of challenge inoculation with *C. gloeosporioides*. After that, the disease aggravated with increased incidence and severity in subsequent days. On day 14, high disease incidence and severity (95% and 90%, respectively) were detected in untreated yam seedlings, and most of the seedlings died rapidly (Figure 3A). However, yam seedlings treated with strain HNM0140^T^ showed a low disease incidence and severity (22% and 20%, respectively) after 14 days (Figures 3B, C). These results indicated that yam seedlings colonized by strain HNM0140^T^ displayed consistent resistance against the pathogen and reduced markedly the anthracnose incidence and severity of yam seedlings. Consequently, strain HNM0140^T^ can be considered as a candidate for the biocontrol of yam anthracnose caused by *C. gloeosporioides*.

### Phylogenetic analyses

**Figure 4:**
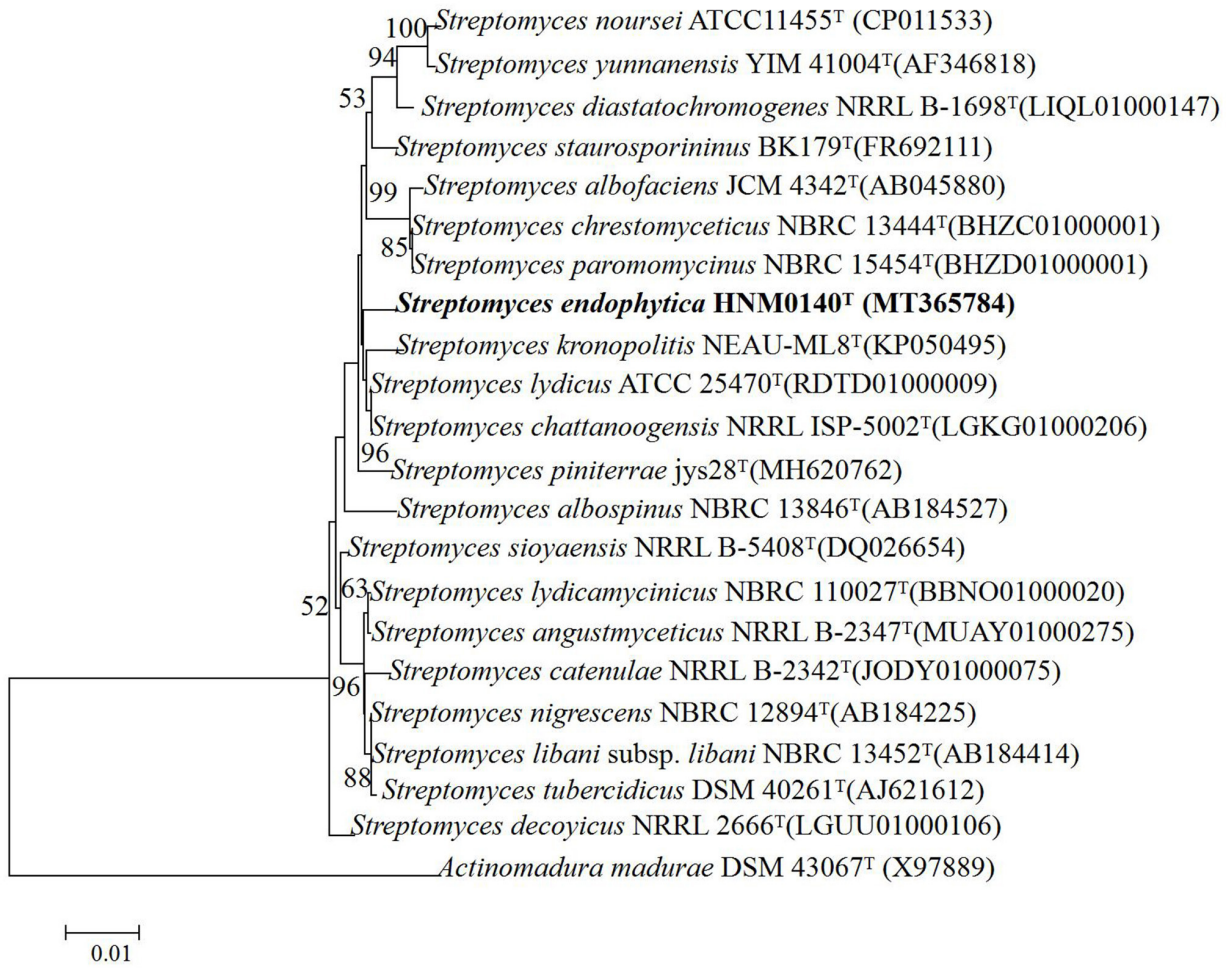
Neighbor-joining phylogenetic tree of strain HNM0140^T^ based on 16S rRNA gene sequences. The bootstrap values (%) presented at the branches were calculated from 1,000 replications, only values >50% are given. Scale bar indicates 0.01 substitutions per nucleotide position.

**Figure 5:**
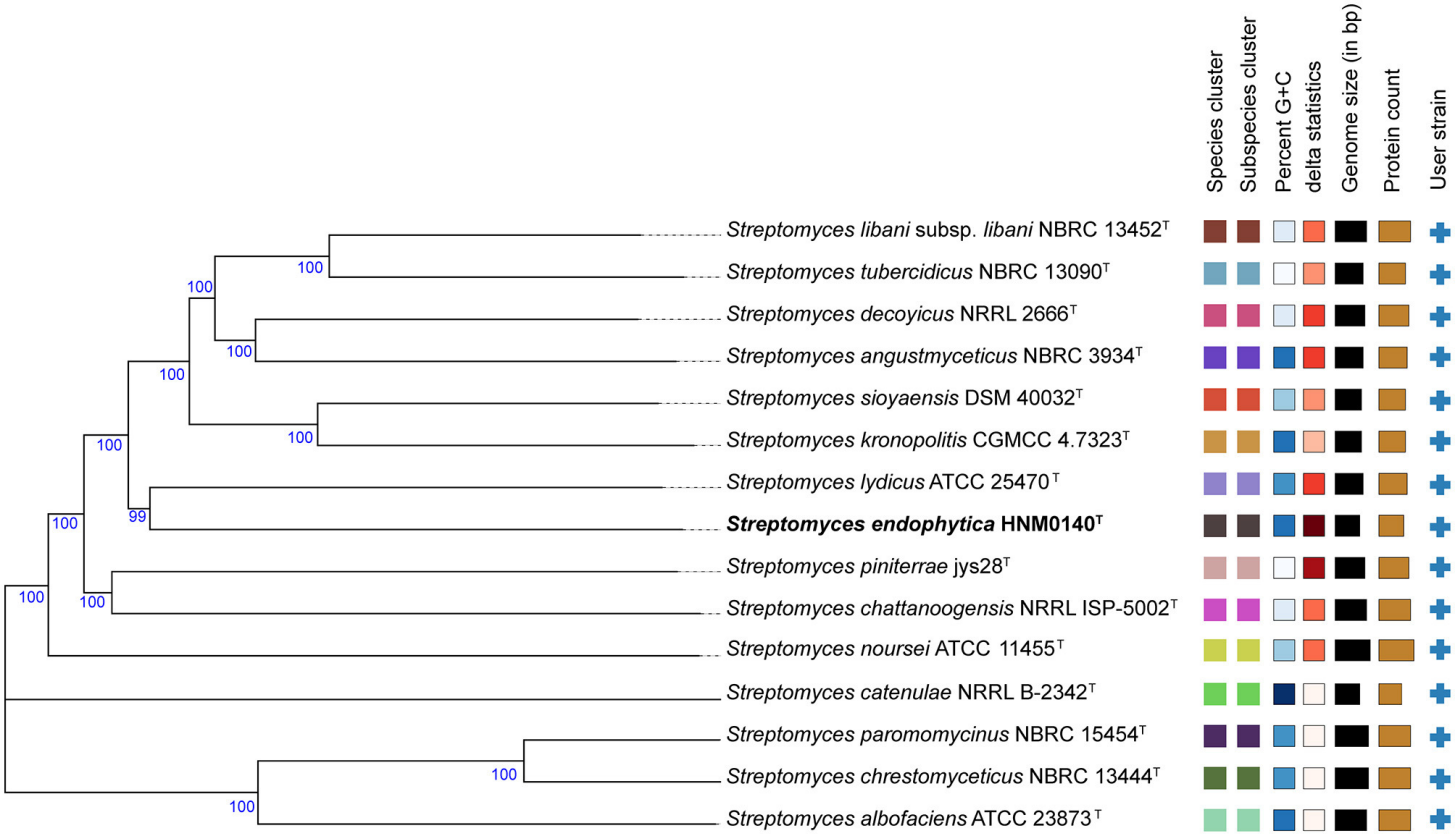
Phylogenomic tree of strain HNM0140^T^ reconstructed on the TYGS (https://tygs.dsmz.de/). Tree inferred with FastME 2.1.6.1 from genome blast distance phylogeny (GBDP) distances calculated from genome sequences. The branch lengths are scaled in terms of the GBDP distance formula d_5_. The numbers above branches are GBDP pseudo-bootstrap support values >60% from 100 replications.

The almost complete 16S rRNA gene sequence (1492 nt, GenBank Accession Number NT365784) of strain HNM0140^T^ was obtained. Based on an EzBioCloud analysis, strain HNM0140^T^ showed the highest similarity to *Streptomyces lydicus* ATCC 25470^T^ (99.45%), *Streptomyces chattanoogensis* NRRL ISP-5002^T^ (99.45%), and *Streptomyces kronopolitis* NEAU-ML8^T^ (98.97%), and <99% similarity to other type species of the genus *Streptomyces*. Phylogenetic analysis (Figure 4; Supplementary Figures 2, 3) indicated that strain HNM0140^T^ formed a separate cluster together with *Streptomyces lydicus* ATCC 25470^T^, *Streptomyces chattanoogensis* NRRL ISP-5002^T^, and *Streptomyces kronopolitis* NEAU-ML8^T^. The phylogenomic tree showed that strain HNM0140^T^ stably clustered with *Streptomyces lydicus* ATCC 25470^T^ (Figure 5). Strain HNM0140^T^ also showed the highest ANI value of 87.05% and the highest dDDH value of 51% with *Streptomyces lydicus* ATCC 25470^T^, while the values of ANI and dDDH between strain HNM0140^T^ and other related type species of the genus *Streptomyces* are shown in Table 2. It is apparent that all values are well below the recommended thresholds (ANI < 95–96% and dDDH < 70%) for species demarcation (Richter and Rosselló-Móra, 2009; Meier-Kolthoff et al., 2013). Therefore, the present genotypic data indicated that strain HNM0140^T^ represented a novel species of the genus *Streptomyces*.

**Table 2:** Average nucleotide identity (ANI) and dDDH values between strain HNM0140^T^ and its closest *Streptomyces* species.

**Type strain**	**HNM0140** ^**T**^	
	**ANI (%)**	**dDDH (%)**
*Streptomyces lydicus* ATCC 25470^T^	87.05	51.0
*Streptomyces libani* subsp. *libani* NBRC 13452^T^	86.49	40.7
*Streptomyces angustmyceticus* NBRC 3934^T^	86.27	41.8
*Streptomyces decoyicus* NRRL 2666^T^	86.43	42.6
*Streptomyces sioyaensis* DSM 40032^T^	86.24	42.1
*Streptomyces tubercidicus* NBRC 13090^T^	85.79	40.2
*Streptomyces kronopolitis* CGMCC 4.7323^T^	85.84	41.0
*Streptomyces chattanoogensis* NRRL ISP-5002^T^	84.66	35.8
*Streptomyces noursei* ATCC 11455^T^	83.94	32.6
*Streptomyces paromomycinus* NBRC 15454^T^	81.56	25.0
*Streptomyces albofaciens* ATCC 23873^T^	81.79	26.0
*Streptomyces chrestomyceticus* NBRC 13444^T^	81.62	25.8
*Streptomyces catenulae* NRRL B-2342^T^	81.62	29.3
*Streptomyces piniterrae* jys 28^T^	84.61	36.3

### Phenotypic analyses

Strain HNM0140^T^ grew well on ISP 1–ISP 6 medium, except for ISP 7 (Supplementary Table S2). The color of aerial hyphae was either white or white gray, while that of substrate mycelia tended to be media dependent. Aerial hyphae further differentiated into long or curl spore chains, showing rugose spore ornamentation (Figures 1C, D). The organism produced no pigment on all test media. Strain HNM0140^T^ was found to survive at pH 3.0–10.0 (optimum pH 7.0) and at 15–40°C (optimum 28°C) and with 0–12% (w/v) NaCl (optimum for 0–5%). The difference in phenotypic properties shown in Table 3 revealed that strain HNM0140^T^ can be clearly distinct from the type strains of its closely related neighbors.

**Table 3:** Differential physiological characteristics between strain HNM0140^T^ and its closely related strains.

**Characteristics**	**1**	**2^a^**	**3^a^**	**4^a^**
**Spore surface**	**Rugose**	**Smooth**	**Spiny**	**Smooth**
**Degradation tests**				
Aesculin	+	+	+	–
Starch	+	–	–	+
Tween 40	+	–	+	+
Production of urease	+	+	+	–
Peptonization and coagulation of milk	–	–	–	+
**Growth on sole carbon sources**				
L-Arabinose	+	+	±	+
D-Mannitol	+	+	±	+
L-Rhamnose	+	±	±	+
D-Sorbitol	+	+	±	+
D-Xylose	+	+	±	+
**Growth on sole nitrogen sources**				
L-Arginine	+	+	±	+
L-Asparagine	+	+	±	+
L-Aspartic acid	+	+	±	+
Creatine	+	+	±	+
Glycine	+	+	±	+
L-Proline	+	+	±	+
L-Serine	+	+	±	±
Growth at pH	3–10	4–8	ND	5–8
Growth at temperature	15–40°C	20–35°C	ND	12–38°C
Growth in NaCl (%, w/v)	0–12	0–2	0–2	0–10

Strains: 1, HNM0140^T^; 2, *S. lydicus* NBRC 13058^T^; 3, *S. chattanoogensis* DSM 40002^T^. 4, *S. kronopolitis* NEAU-ML8^T^. +, Positive; ±, moderately positive; –, negative, ND, not determined.

a Data for reference strains were taken from Liu et al. (2016).

### Chemotaxonomic analyses

LL-Diaminopimelic acid was detected in the cell wall of strain HNM0140^T^ and the cell sugars, namely, glucose and ribose were detected in its whole-organism hydrolysates. Polar lipid analysis revealed that the polar lipid profiles of strain HNM0140^T^ contained phosphatidylmethylethanolamine, diphosphatidylglycerol, phosphatidylinositol mannoside, phosphatidylethanolamine, and one unidentified phospholipid (Supplementary Figure 4). The menaquinone profiles of strain HNM0140^T^ were identified as MK-9(H_6_) (46.3%), MK-9(H_4_) (40.3%), MK-9(H_8_) (8.8%), and MK-9(H_11_) (4.6%). The fatty-acid profiles of strain HNM0140^T^ comprised iso-C_16:0_ (32.2%), C_16:0_ (16.9%), and anteiso-C_15:0_ (12.4%) as its major compositions, and minor amounts (<10%) of anteiso-C_17:0_, iso-C_15:0_, iso-C_14:0_, iso-C_18:0_, and C_17:0_ were also detected. All the aforementioned chemotaxonomic characteristics were consistent with its assignment to the genus *Streptomyces*.

In conclusion, a combination of phylogenetic, genomic, phenotypic, and chemotaxonomic analyses mentioned previously clearly indicated that strain HNM0140^T^ should represent a new streptomycete species, for which the name proposed is *Streptomyces endophytica* sp. nov.

### Genome properties and analyses

The complete genome of strain HNM0140^T^ consisted of one linear chromosome (6,901,780 bp) with G+C content of 72.2%, 6,056 protein-coding genes (CDS), 66 tRNA genes, 18 rRNA genes, and 3 ncRNA genes. Among all CDS, the number of genes annotated into the COG, GO, and KEGG functional categories was 3,916, 1,562, and 2,126, respectively (Table 4). Based on the antiSMASH 6.0 analysis, strain HNM0140^T^ was found to possess 18 putative biosynthetic gene clusters (BGCs) for secondary metabolites, which were distributed across 13 different types, including three butyrolactone, two RiPP-like, two siderophore, two terpene, one ectoine, one lanthipeptide-class-iii, one lassopeptide, one linaridin, one other, and four hybrid gene clusters (Supplementary Table S3). Seven putative BGCs showed at least 50% similarity to known BGCs, including three BGCs encoding for ectoine, desferrioxamine E, and althiomycin with 100% similarity. Among them, ectoine was an osmotic and cold stress protectant produced by *Streptomyces* (Prabhu et al., 2004). Desferrioxamine E is a cyclic trihydroxamate siderophore that exhibits antimicrobial activity against *Mycobacterium* species by inhibiting biofilm formation (Ishida et al., 2011). Althiomycin is a broad-spectrum antibiotic that displays antibacterial activity against both Gram-negative and -positive bacteria by inhibiting protein synthesis (Fujimoto et al., 1970). Other putative BGCs showed a low similarity (<30%) to known BGCs, including two BGCs with no similarity, which highlighted the potential of strain HNM0140^T^ to produce novel antimicrobial metabolites.

**Table 4:** Genome features of strain HNM0140^T^.

**Feature**	**Chromosome characteristics**
**Genome topology**	**Linear**
Chromosome size (bp)	6,901,780
GC content	72.2%
Protein-coding genes	6,056
Gene average length (bp)	998
Genes assigned to COG	3,916
Genes assigned to GO	1,562
Genes assigned to KEGG	2,126
rRNA genes	18
tRNA genes	66
ncRNA genes	3
Secondary metabolites clusters	18

Genome annotation and analysis uncovered that strain HNM0140^T^ possessed a series of genes related to PGP, such as N2 fixation, production of IAA, phosphate solubilization, siderophore, and production of ACC deaminase (Supplementary Table S4). The genome of strain HNM0140^T^ harbors a *nifU* gene, which encodes a protein (NifU) involved in the synthesis of iron–sulfur metalloclusters that are thought to be required for nitrogen fixation (Hwang et al., 1996). Strain HNM0140^T^ also contains an *iaaM* gene that potentially participated in the indole-3-acetamide pathway of the indole-3-acetic acid biosynthesis (Qin et al., 2017). The genome of strain HNM0140^T^ carries multiple genes that were related to the transport and solubilization of inorganic polyphosphates, including two *gdh* genes and a *pqqD* gene that participated in the synthesis of gluconic acid, which was believed to be responsible for the degradation of mineral phosphates (Farhat et al., 2015), and two *pit* genes, five *phn* genes, and three *pst* genes that participated in the transport of phosphonates. Strain HNM0140^T^ also harbors plenty of *rhbCDEF* genes involved in the synthesis of the siderophores. Moreover, two BGCs encoding for siderophores, such as desferrioxamine E and ficellomycin, were predicted to be in the genome of strain HNM0140^T^ (Supplementary Table S3). The *acdS* gene coding for ACC deaminase was also found in the genome, which has been reported to promote plant growth in stress conditions by decreasing ethylene concentrations (Santoyo et al., 2016). In addition, several gene coding for antifungal enzymes, such as β-glucanase and chitinase, were also present in the genome, indicating the potential inhibitory mechanism of strain HNM0140^T^ against *C. gloeosporioides*.

### Description of *Streptomyces endophytica* sp. nov.

*Streptomyces endophytica* (en.do.phy′ti.ca. Gr. pref. *endo* within; Gr. neut. n. *phyton* plant; L. fem. suff. -*ica* adjectival suffix used with the sense of belonging to; N.L. fem. adj. *endophytica* within plant, endophytic, pertaining to the original isolation from plant tissues).

Gram-stain-positive, aerobic and non-motile actinomycete, which forms an extensively branched substrate and aerial hyphae that differentiate into long or curl spore chains, consisting of rugose spores at maturity, grows well on ISP1–ISP6 media. No diffusible pigments were observed on ISP1–ISP7 agars. The growth occured at pH 3.0–10.0 (optimum pH 7.0), at 15–40°C (optimum 28°C), and up to 12% (w/v) NaCl tolerance (optimum 0–5%). It was positive for hydrolysis of starch, aesculin, Tween 40 and production of urease but negative for milk peptonization and coagulation. D-Xylose, L-arabinose, raffinose, D-mannitol, L-rhamnose, myo-inositol, fructose, raffinose, sucrose, D-galactose, and D-glucose are utilized as sole carbon sources. L-Aspartic acid, glycine, L-arginine, creatine, L-asparagine, L-proline, and L-serine are utilized as sole nitrogen sources.

The cell wall of strain HNM0140^T^ consists of LL-diaminopimelic acid. MK-9(H_6_) and MK-9(H_4_) are its major menaquinones, and minor amounts of MK-9(H_8_) and MK-9(H_11_) were also detected. Iso-C_16:0_, C_16:0_, and anteiso-C_15:0_ are its major fatty-acid compositions, and minor amounts of anteiso-C_17:0_, iso-C_15:0_, iso-C_14:0_, iso-C_18:0_, and C_17:0_ were also detected. The major phospholipids comprise phosphatidylmethylethanolamine, diphosphatidylglycerol, phosphatidylinositol mannoside, and phosphatidylethanolamine.

The type strain, HNM0140^T^ (=CCTCC AA2019073^T^ = LMG 31910^T^), was isolated from the root of yam collected from Danzhou City of Hainan Province, China. The 16S rRNA gene sequence of strain HNM0140^T^ was deposited in the GenBank/EMBL/DDBJ (accession no. MT365784). The complete genome of HNM0140^T^ consists of 6,901,780 bp and the DNA G+C content of the type strain is 72.2% mol%. The complete genome sequence of HNM0140^T^ is available in GenBank under Accession Number CP110636.

## Conclusion

A new endophytic actinobacterium strain HNM0140^T^ was isolated from the root of yam (*Dioscorea opposita* Thunb.) and selected for its strong antifungal activity against *C. gloeosporioides*. Strain HNM0140^T^ was identified as a novel *Streptomyces* species through polyphasic taxonomic analyses, and its name was proposed as *Streptomyces endophytica* sp. nov. The organism could colonize in the tissue-cultured seedlings of yam and markedly reduce the severity and incidence of yam anthracnose under greenhouse conditions. The whole genome of strain HNM0140^T^ harbored 18 BGCs for secondary metabolites, PGP-related genes, and several genes coding for antifungal enzymes. Therefore, strain HNM0140^T^ will be a promising candidate for the biocontrol of yam anthracnose.

## Data availability statement

The datasets presented in this study can be found in online repositories. The names of the repository/repositories and accession number(s) can be found in the article/Supplementary material.

## Author contributions

SZ and XH conceived and designed the study. SZ and YZ carried out all the experiments. CL, WW, YX, WX, and DH conducted the data analysis. SZ prepared the manuscript. XH revised it. All authors reviewed and approved the manuscript.
