# Supplementary material for: Identification and genomic analyses of a novel endophytic actinobacterium Streptomyces endophytica sp. nov. with potential for biocontrol of yam anthracnose
Source: Front Microbiol. 2023 Apr 4;14:1139456. doi: 10.3389/fmicb.2023.1139456 (PMC10111032; doi:10.3389/fmicb.2023.1139456)
Supplement: Supplementary file 1 [file Data_Sheet_1.docx]

**Supplementary Material**

**Identification and genomic analyses of a novel endophytic actinobacterium** ***Streptomyces endophytica* sp*.* nov*.* with potential for biocontrol of yam anthracnose**

Shuangqing Zhou^1,2^, Yifan Zhou^3^, Chengui Li^2^, Wenqiang Wu^2^, Yun Xu^2^,Wei Xia^3^, Dongyi Huang^3^, Xiaolong Huang^2^*

^1^ Department of Pharmacognosy, College of Pharmacy, Guilin Medical University, Guilin, 541199, PR China.

^2^ Department of Biotechnology, School of Life Sciences, Hainan University, Haikou 570228, PR China

^3^ Department of Agronomy, College of Tropical Crops, Hainan University, Haikou 570228, PR China

^*^Corresponding author, Phone: +86-898-36323319, Fax: +86-898-36323319, E-mail: huangxialong@hainanu.edu.cn.

Address: School of Life Sciences, Hainan University, Haikou, 570228, PR China.

**Table S1.** **Inhibition zone of 23 tested endophytic actinomycetes against *C. gloeosporioides.***

^a^,Values represent mean width of growth inhibition zones; ^b^, Average ± standard error (three replicates).

| Isolates | Source | Width of growth |
| --- | --- | --- |
|  |  | inhibition zone (mm)^a^ |
| HNM0131 | Root | 9.2±0.1^b^ |
| HNM0140 | Root | 24.3±0.2 |
| HNM0133 | Root | 3.2±0.1 |
| HNM0134 | Root | 5.5±0.1 |
| HNM0136 | Root | 8.5±0.2 |
| HNM0310 | Root | 5.7±0.1 |
| HNM0311 | Root | 9.6±0.1 |
| HNM0312 | Root | 6.9±0.3 |
| HNM0315 | Root | 12.4±0.1 |
| HNM0316 | Root | 3.1±0.1 |
| HNM0321 | Root | 4.3±0.4 |
| HNM0322 | Root | 14.3±0.1 |
| HNM0326 | Root | 4.6±0.1 |
| HNM0334 | Root | 16.6±0.2 |
| HNM0343 | Root | 12.7±0.1 |
| HNM0344 | Root | 14.2±0.3 |
| HNM0345 | Root | 10.1±0.1 |
| HNM0346 | Root | 5.4±0.1 |
| HNM0350 | Root | 13.8±0.1 |
| HNM0360 | Root | 10.2±0.1 |
| HNM0365 | Root | 6.3±0.2 |
| HNM0376 | Root | 9.6±0.1 |
| HNM0386 | Root | 5.2±0.1 |

**Table S2. Growth and cultural characteristics of strain HNM0140^T^.**

^a^, ISP1:Tryptone-yeast extract agar; ISP2: Yeast extract - malt extract agar；ISP3:Oatmeal agar; ISP4: Inorganic salts - starch agar; ISP5: Glycerol - asparagine agar; ISP6: Peptone - yeast extract iron agar; ISP7: Tyrosine agar.
^b^, +++, Good growth; ++, moderate growth; +, poor growth.

| Agar medium^a^ | Growth^b^ | Aerial mycelium | Substrate mycelium | Soluble pigment |
| --- | --- | --- | --- | --- |
| ISP1 | +++ | White gray | Light yellow | none |
| ISP2 | +++ | White gray | Light yellow | none |
| ISP3 | ++ | white | Grayish yellow | none |
| ISP4 | +++ | white | Light yellowish brown | none |
| ISP5 | +++ | white | Grayish yellow | none |
| ISP6 | ++ | white | Grayish yellow | none |
| ISP7 | + | white | Dark orange yellow | none |

**Table S3.** Annotation results of secondary metabolite gene cluster from strain HNM0140^T^

| Cluster ID | Type | From | To | Most similar known cluster | similarity |
| --- | --- | --- | --- | --- | --- |
| [Cluster1](https://antismash.secondarymetabolites.org/upload/bacteria-7b2a1717-35a9-48a7-9eb8-79ca9c2cb9d6/index.html#r1c1) | [butyrolactone](https://docs.antismash.secondarymetabolites.org/glossary/#butyrolactone) | 27,067 | 36,744 | [auroramycin](https://mibig.secondarymetabolites.org/go/BGC0001522/1) | 5% |
| [Cluster2](https://antismash.secondarymetabolites.org/upload/bacteria-7b2a1717-35a9-48a7-9eb8-79ca9c2cb9d6/index.html#r1c1) | [siderophore](https://docs.antismash.secondarymetabolites.org/glossary/#siderophore) | 92,939 | 104,162 | [ficellomycin](https://mibig.secondarymetabolites.org/go/BGC0001593/1) | 3% |
| [Cluster3](https://antismash.secondarymetabolites.org/upload/bacteria-7b2a1717-35a9-48a7-9eb8-79ca9c2cb9d6/index.html#r1c1) | [linaridin](https://docs.antismash.secondarymetabolites.org/glossary/#linaridin) | 1,569,177 | 1,590,106 | [legonaridin](https://mibig.secondarymetabolites.org/go/BGC0001188/1) | 66% |
| [Cluster4](https://antismash.secondarymetabolites.org/upload/bacteria-7b2a1717-35a9-48a7-9eb8-79ca9c2cb9d6/index.html#r1c1) | [terpene](https://docs.antismash.secondarymetabolites.org/glossary/#terpene) | 1,726,307 | 1,744,991 | [salinomycin](https://mibig.secondarymetabolites.org/go/BGC0000144/1) | 6% |
| [Cluster5](https://antismash.secondarymetabolites.org/upload/bacteria-7b2a1717-35a9-48a7-9eb8-79ca9c2cb9d6/index.html#r1c1) | [lanthipeptide-class-iii](https://docs.antismash.secondarymetabolites.org/glossary/#lanthipeptide-class-iii) | 2,126,316 | 2,148,997 | [phthoxazolin](https://mibig.secondarymetabolites.org/go/BGC0001740/1) | 4% |
| [Cluster6](https://antismash.secondarymetabolites.org/upload/bacteria-7b2a1717-35a9-48a7-9eb8-79ca9c2cb9d6/index.html#r1c1) | [lassopeptide](https://docs.antismash.secondarymetabolites.org/glossary/#lassopeptide) | 3,003,377 | 3,026,454 | [moomysin](https://mibig.secondarymetabolites.org/go/BGC0001673/1) | 50% |
| [Cluster7](https://antismash.secondarymetabolites.org/upload/bacteria-7b2a1717-35a9-48a7-9eb8-79ca9c2cb9d6/index.html#r1c1) | [ectoine](https://docs.antismash.secondarymetabolites.org/glossary/#ectoine) | 4,186,241 | 4,196,657 | [ectoine](https://mibig.secondarymetabolites.org/go/BGC0000853/1) | 100% |
| [Cluster8](https://antismash.secondarymetabolites.org/upload/bacteria-7b2a1717-35a9-48a7-9eb8-79ca9c2cb9d6/index.html#r1c1) | [siderophore](https://docs.antismash.secondarymetabolites.org/glossary/#siderophore) | 4,279,463 | 4,291,253 | [desferrioxamine E](https://mibig.secondarymetabolites.org/go/BGC0001478/1) | 100% |
| [Cluster9](https://antismash.secondarymetabolites.org/upload/bacteria-7b2a1717-35a9-48a7-9eb8-79ca9c2cb9d6/index.html#r1c1) | thioamide-NRP,NRPS | 4,720,200 | 4,781,376 | [ishigamide](https://mibig.secondarymetabolites.org/go/BGC0001623/1) | 50% |
| [Cluster10](https://antismash.secondarymetabolites.org/upload/bacteria-7b2a1717-35a9-48a7-9eb8-79ca9c2cb9d6/index.html#r1c1) | NRPS,T1PKS,T2PKS | 5,058,016 | 5,170,825 | [althiomycin](https://mibig.secondarymetabolites.org/go/BGC0000955/1) | 100% |
| [Cluster11](https://antismash.secondarymetabolites.org/upload/bacteria-7b2a1717-35a9-48a7-9eb8-79ca9c2cb9d6/index.html#r1c1) | RiPP-like,NRPS | 5,211,686 | 5,318,691 | [mannopeptimycin](https://mibig.secondarymetabolites.org/go/BGC0000388/1) | 7% |
| [Cluster12](https://antismash.secondarymetabolites.org/upload/bacteria-7b2a1717-35a9-48a7-9eb8-79ca9c2cb9d6/index.html#r1c1) | [other](https://docs.antismash.secondarymetabolites.org/glossary/#other) | 5,370,270 | 5,411,155 | [A-503083 A / B / E / F](https://mibig.secondarymetabolites.org/go/BGC0000288/1) | 7% |
| [Cluster13](https://antismash.secondarymetabolites.org/upload/bacteria-7b2a1717-35a9-48a7-9eb8-79ca9c2cb9d6/index.html#r1c1) | [butyrolactone](https://docs.antismash.secondarymetabolites.org/glossary/#butyrolactone) | 5,550,121 | 5,559,769 | [herboxidiene](https://mibig.secondarymetabolites.org/go/BGC0001065/1) | 2% |
| [Cluster14](https://antismash.secondarymetabolites.org/upload/bacteria-7b2a1717-35a9-48a7-9eb8-79ca9c2cb9d6/index.html#r1c1) | [RiPP-like](https://docs.antismash.secondarymetabolites.org/glossary/#ripp-like) | 5,588,861 | 5,599,211 | [jomthonic acid A / B / C](https://mibig.secondarymetabolites.org/go/BGC0001457/1) | 5% |
| [Cluster15](https://antismash.secondarymetabolites.org/upload/bacteria-7b2a1717-35a9-48a7-9eb8-79ca9c2cb9d6/index.html#r1c1) | [terpene](https://docs.antismash.secondarymetabolites.org/glossary/#terpene) | 6,122,617 | 6,149,284 | [hopene](https://mibig.secondarymetabolites.org/go/BGC0000663/1) | 69% |
| [Cluster16](https://antismash.secondarymetabolites.org/upload/bacteria-7b2a1717-35a9-48a7-9eb8-79ca9c2cb9d6/index.html#r1c1) | [RiPP-like](https://docs.antismash.secondarymetabolites.org/glossary/#ripp-like) | 6,710,937 | 6,721,800 | - | - |
| [Cluster17](https://antismash.secondarymetabolites.org/upload/bacteria-7b2a1717-35a9-48a7-9eb8-79ca9c2cb9d6/index.html#r1c1) | [butyrolactone](https://docs.antismash.secondarymetabolites.org/glossary/#butyrolactone) | 6,796,447 | 6,807,430 | - | - |
| [Cluster18](https://antismash.secondarymetabolites.org/upload/bacteria-7b2a1717-35a9-48a7-9eb8-79ca9c2cb9d6/index.html#r1c1) | NRPS,other,T3PKS | 6,821,220 | 6,882,326 | [lipopeptide 8D1-1 / 8D1-2](https://mibig.secondarymetabolites.org/go/BGC0001370/1) | 18% |

**Table S4. annotation results of growth promoting related functional genes of strain HNM0140^T^.**

| Gene ID | Gene name | Gene annotation |
| --- | --- | --- |
| GE03600 | *nifU* | nitrogen fixation protein NifU |
| GE00620 | *acdS* | 1-aminocyclopropane-1-carboxylate deaminase |
| GE04117 | *iaaM* | Tryptophan 2-monooxygenase |
| GE02640 | *pqqD* | Coenzyme PQQ synthesis protein D (PqqD) |
| GE00367 | *gdh* | NADP-specific glutamate dehydrogenase |
| GE02607 | *gdhB* | NAD-specific glutamate dehydrogenase |
| GE01277 | *pit* | low-affinity inorganic phosphate transporter |
| GE03648 | *pit* | low-affinity inorganic phosphate transporter |
| GE02729 | *PhnT* | 2-aminoethylphosphonate import ATP-binding protein |
| GE02731 | *PhnV* | 2-aminoethylphosphonate transport system permease protein PhnV |
| GE02730 | *PhnU* | 2-aminoethylphosphonate transport system permease protein PhnU |
| GE02732 | *phnS* | 2-aminoethylphosphonate-binding periplasmic protein phnS |
| GE00803 | *PhnC* | Phosphonates import ATP-binding protein PhnC |
| GE01276 | *PstB* | Phosphate import ATP-binding protein PstB |
| GE02962 | *pstS1* | Phosphate-binding protein PstS 1 (Precursor) GN=pstS1 |
| GE01273 | *pstS2* | Phosphate-binding protein PstS 3 (Precursor) GN=pstS2 |
| GE03516 | rhbE | Rhizobactin siderophore biosynthesis protein RhbE |
| GE03719 | rhbF | Rhizobactin siderophore biosynthesis protein RhbF |
| GE00085 | rhbF | Rhizobactin siderophore biosynthesis protein RhbF |
| GE00087 | rhbC | Rhizobactin siderophore biosynthesis protein RhbC |
| GE03720 | rhbD | Rhizobactin siderophore biosynthesis protein RhbD |
| GE03741 | glucan | 1,3-beta-glucanase |
| GE04251 | bglA | Beta-glucanase |
| GE04985 | chiC | Chitinase C |
| GE00354 | chi01 | Exochitinase 1 |
| GE01894 | chiA2 | Chitinase |
| GE00831 | chiD | Chitinase D |

**Figure S1. In vitro growth promotion of Arabidopsis seedlings by strain HNM0140^T^.**

(A) The entire seedlings co-cultured with HNM0140^T^ or non-treatment (control) for a week, (B) The root of seedlings in non-treatment , (C) The root of seedlings in HNM0140^T^ treatment. The pictures that showed the amount of root hairs with a methylene blue stain were taken using a light microscopy at 20× magnification. (D-E) The primary root length and fresh weight of seedling co-cultured with HNM0140^T^ or non-treatment (control) for a week. Data are shown as mean ± SE (*n* ≥20; **p*≤0.05, Student's *t*-test)


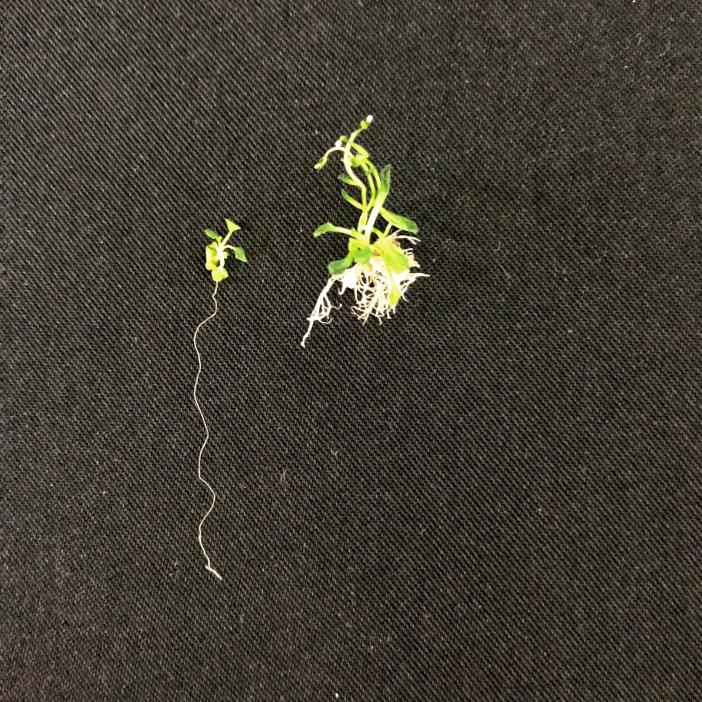


**Control**

(A)


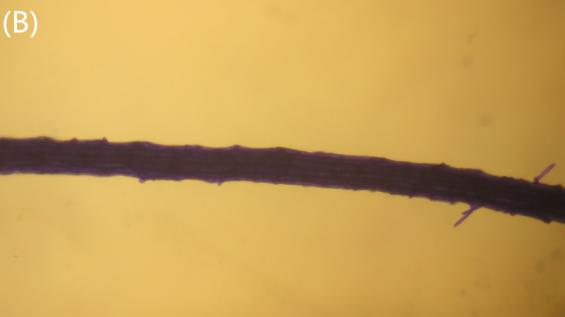

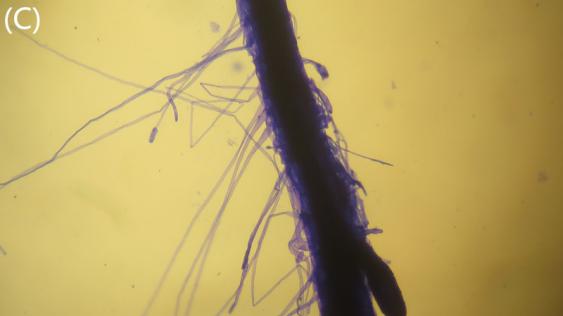


**HNM0140**

(E)

(D)

**Figure S2.** **Minimum Evolution phylogenetic tree of strain HNM0140^T^ based on 16S rRNA gene sequences.** The bootstrap values (%) presented at the branches were calculated from 1000 replications, only values > 50% are given. Scale bar indicates 0.01 substitutions per nucleotide position.

*Streptomyces noursei* ATCC 11455^T^(CP011533)

*Streptomyces yunnanensis* YIM 41004 ^T^ (AF346818)

*Streptomyces diastatochromogenes* NRRL B-1698 ^T^ (LIQL01000147)

*Streptomyces staurosporininus* BK179 ^T^ (FR692111)

*Streptomyces piniterrae* jys28 ^T^ (MH620762)

*Streptomyces albospinus* NBRC 13846 ^T^ (AB184527)

***Streptomyces endophytica* HNM0140^T^ (MT365784)**

*Streptomyces kronopolitis* NEAU-ML8 ^T^ (KP050495)

*Streptomyces lydicus* ATCC 25470 ^T^(RDTD01000009)

*Streptomyces chattanoogensis* NRRL ISP-5002 ^T^ (LGKG01000206)

*Streptomyces albofaciens* JCM 4342 ^T^(AB045880)

*Streptomyces chrestomyceticus* NBRC 13444 ^T^ (BHZC01000001)

*Streptomyces paromomycinus* NBRC 15454 ^T^(BHZD01000001)

*Streptomyces sioyaensis* NRRL B-5408 ^T^ (DQ026654)

*Streptomyces catenulae* NRRL B-2342 ^T^ (JODY01000075)

*Streptomyces lydicamycinicus* NBRC 110027 ^T^ (BBNO01000020)

*Streptomyces angustmyceticus* NRRL B-2347 ^T^ (MUAY01000275)

*Streptomyces libani* subsp. *libani* NBRC 13452 ^T^ (AB184414)

*Streptomyces nigrescens* NBRC 12894 ^T^ (AB184225)

*Streptomyces tubercidicus* DSM 40261 ^T^ (AJ621612)

*Streptomyces decoyicus* NRRL 2666 ^T^ (LGUU01000106)

*Actinomadura madurae* DSM 43067^T^ (X97889)

100

85

99

95

89

74

95

59

98

0.01

**Figure S3. Maximum-Likelihood phylogenetic tree of strain HNM0140^T^ based on 16S rRNA gene sequences.** The bootstrap values (%) presented at the branches were calculated from 1000 replications, only values > 50% are given. Scale bar indicates 0.01 substitutions per nucleotide position.

*Streptomyces noursei* ATCC 11455^T^(CP011533)

*Streptomyces yunnanensis* YIM 41004^T^(AF346818)

*Streptomyces diastatochromogenes* NRRL B-1698^T^(LIQL01000147)

*Streptomyces staurosporininus* BK179^T^(FR692111)

*Streptomyces albofaciens* JCM 4342^T^(AB045880)

*Streptomyces chrestomyceticus* NBRC 13444^T^(BHZC01000001)

*Streptomyces paromomycinus* NBRC 15454^T^(BHZD01000001)

*Streptomyces lydicus* ATCC 25470^T^(RDTD01000009)

*Streptomyces chattanoogensis* NRRL ISP-5002^T^(LGKG01000206)

*Streptomyces kronopolitis* NEAU-ML8^T^(KP050495)

***Streptomyces endophytica* HNM0140^T^ (MT365784)**

*Streptomyces piniterrae* jys28^T^(MH620762)

*Streptomyces lydicamycinicus* NBRC 110027^T^(BBNO01000020)

*Streptomyces angustmyceticus* NRRL B-2347^T^(MUAY01000275)

*Streptomyces catenulae* NRRL B-2342^T^(JODY01000075)

*Streptomyces nigrescens* NBRC 12894^T^(AB184225)

*Streptomyces libani* subsp. *libani* NBRC 13452^T^(AB184414)

*Streptomyces tubercidicus* DSM 40261^T^(AJ621612)

*Streptomyces sioyaensis* NRRL B-5408^T^(DQ026654)

*Streptomyces albospinus* NBRC 13846^T^(AB184527)

*Streptomyces decoyicus* NRRL 2666^T^(LGUU01000106)

*Actinomadura madurae* DSM 43067^T^ (X97889)

99

96

92

85

94

55

0.01

**Figure S4. Major polar lipids of strain HNM0140^T^**

Abbreviations: DPG, diphosphatidylglycerol; PME, phosphatidylmethylethanolamine; PE, phosphatidylethanolamine; PIM, phosphatidylinositol mannoside; PL, unidentiﬁed phospholipid.


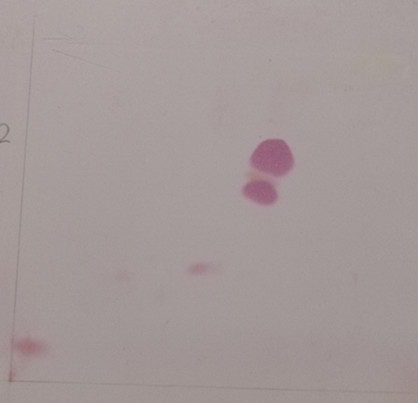

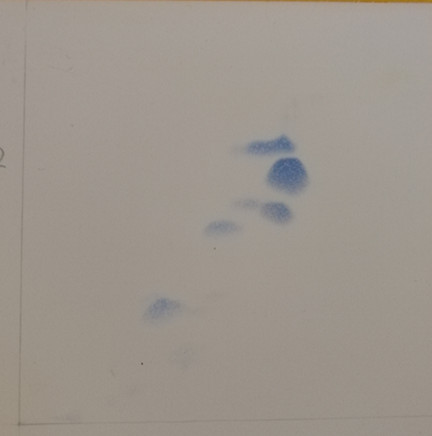


PE

PME

PL

PIM

PE

PME

DPG
